# Nifedipine induced gingival enlargement in an edentulous patient: a case report with one year follow up

**DOI:** 10.1186/s12903-018-0690-4

**Published:** 2018-12-27

**Authors:** Shaik Mohammed Asif, Naheeda Shaik, Bhavna Barthunia, Sultan Mohammed Kaleem, M Zakirulla, Mohammed Zahir Kota, Fawaz Abdul Hamid Baig

**Affiliations:** 1 Department of Diagnostic Sciences and Oral biology , College of Dentistry, King Khalid University, Abha, Kingdom of Saudi Arabia; 2 Department of Periodontology, Mamata Dental College, Khammam, India; 3 Department of Oral Medicine and Radiology, Daswani Dental College and Research Hospital, Kota, Rajasthan India; 4 Department of Diagnostic Sciences and Oral Biology, College of Dentistry, King Khalid University, Abha, Kingdom of Saudi Arabia; 5 Department of Pediatric Dentistry and Orthodontic Sciences, College of Dentistry, King Khalid University, Abha, Kingdom of Saudi Arabia; 6 Department of Oral and Maxillofacial Surgery, College of Dentistry, King Khalid University, Abha, Kingdom of Saudi Arabia

**Keywords:** Nifedipine induced, Edentulous patient, Gingival enlargement

## Abstract

**Background:**

Gingival enlargement due to calcium channel blockers is a common complaint reported by patients. It can be localized or generalized and can range from mild to severe, affecting patients appearance and function. Nifedipine induced gingival enlargement is noticed only in 10 % of patients and very few cases of Nifedipine induced gingival enlargement in an edentulous patient have been documented in the literature.

**Case presentation:**

Here in, we report a case of gingival enlargement in a 70 year old hypertensive edentulous patient who was on low dose Nifedipine therapy. Patient wanted complete dentures. We planned to excise the overgrowth and followed up for 1 year.

**Conclusion:**

Nifedipine induced gingival enlargement noticed only in 10 % of patients. Hence, there is a need for physicians and dentist to make a coordinated treatment plan and practice care while prescribing these drugs which are associated with gingival overgrowth.

## Background

Gingival hyperplasia is a multifactorial disease and drug induced gingival hyperplasia is an esthetically disfiguring over growth attributable to various medications [1, 2]. Calcium channel blockers (CCBs) are the most commonly prescribed anti-hypertensive drugs for patients with cardiovascular disorders. Gingival hyperplasia on long term use of Nifedipine is rare in the literature [3]. The first documented case of Nifedipine induced gingival enlargement was reported in 1984 [4].Clinically-evident overgrowth of gingiva can be seen within 1–2 months after initiation of therapy. Incidence rate of nifedipine- induced gingival enlargement is 5–10% [5]. Various factors attribute for overgrowth of gingiva, which include poor oral hygiene, genetic factors, individual susceptibility, and interaction between drugs and its metabolites with fibroblast of gingiva [6]. Moreover age and gender have also been considered as risk factors for drug induced gingival enlargement [4, 7]. Nifedipine-induced gingival enlargement in an edentulous patient is rare in literature. Therefore, herein, we report a case of nifedipine-induced gingival enlargement in an edentulous patient.

## Case presentation

**Fig. 1:**
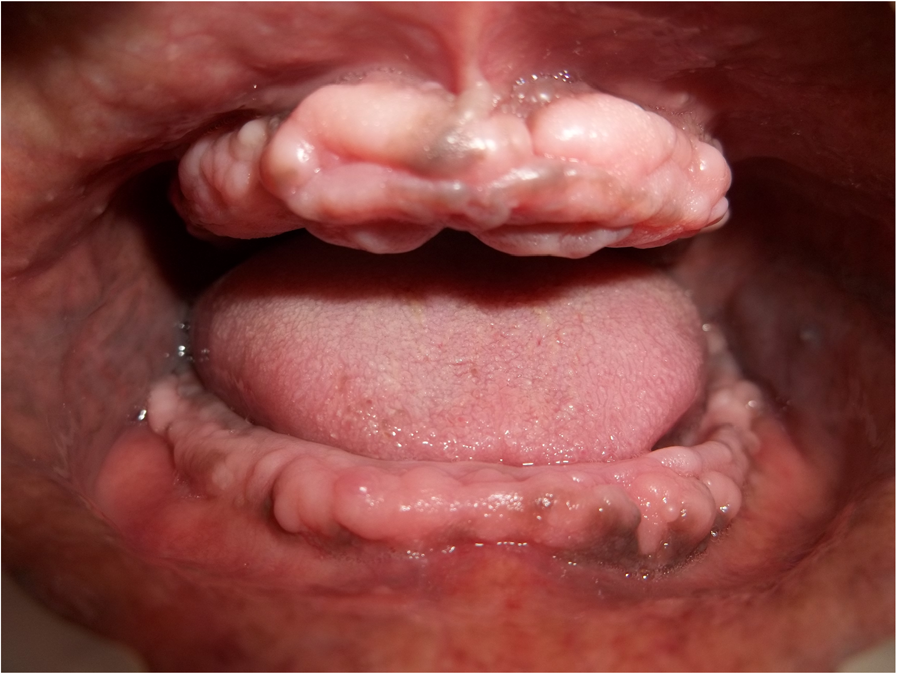
Gingival enlargement in edentulous ridges

**Fig. 2:**
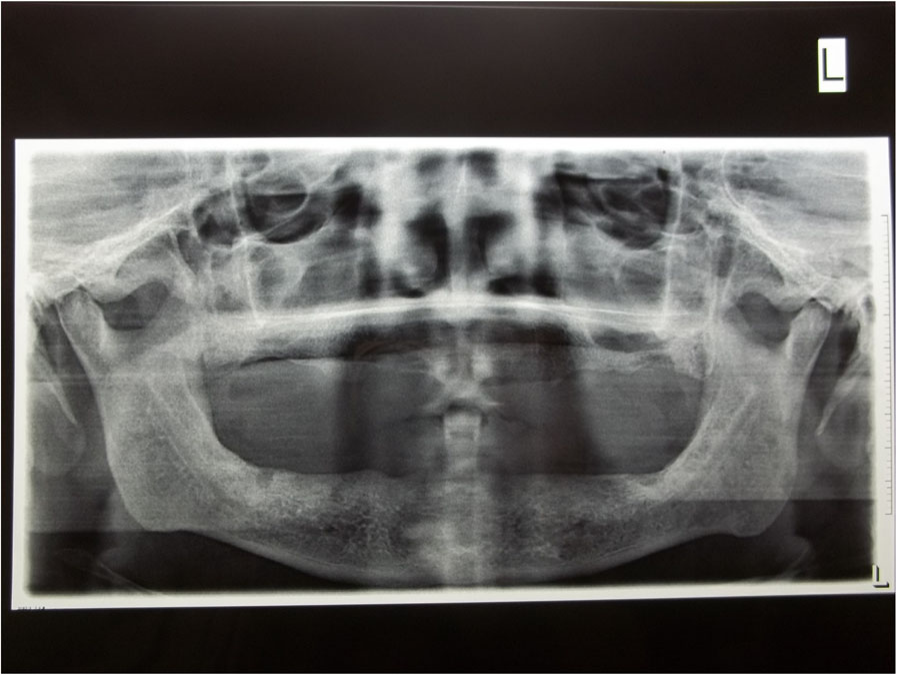
Panoramic radiograph shows no osseous deformities seen in maxilla and mandible

**Fig. 3:**
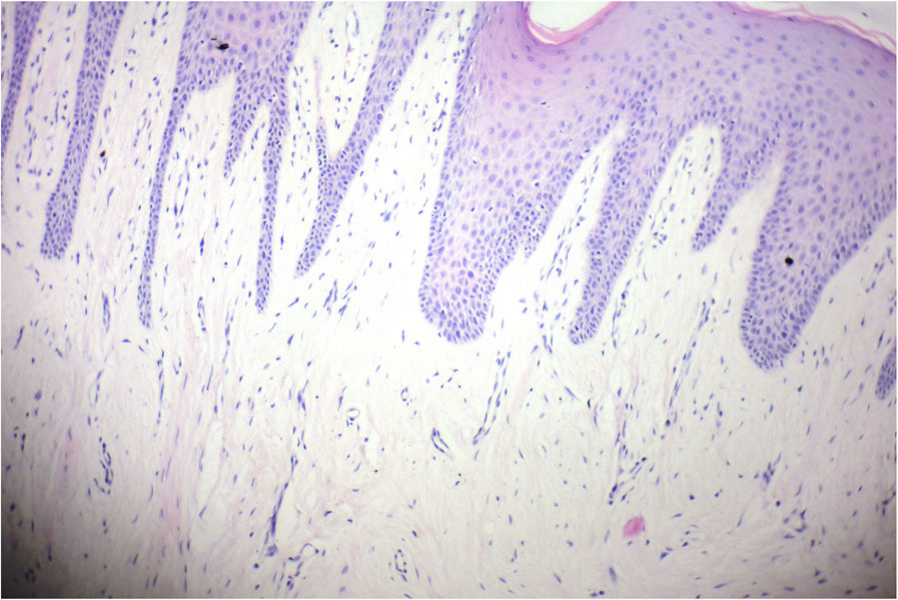
Histological picture of excised tissue

**Fig. 4:**
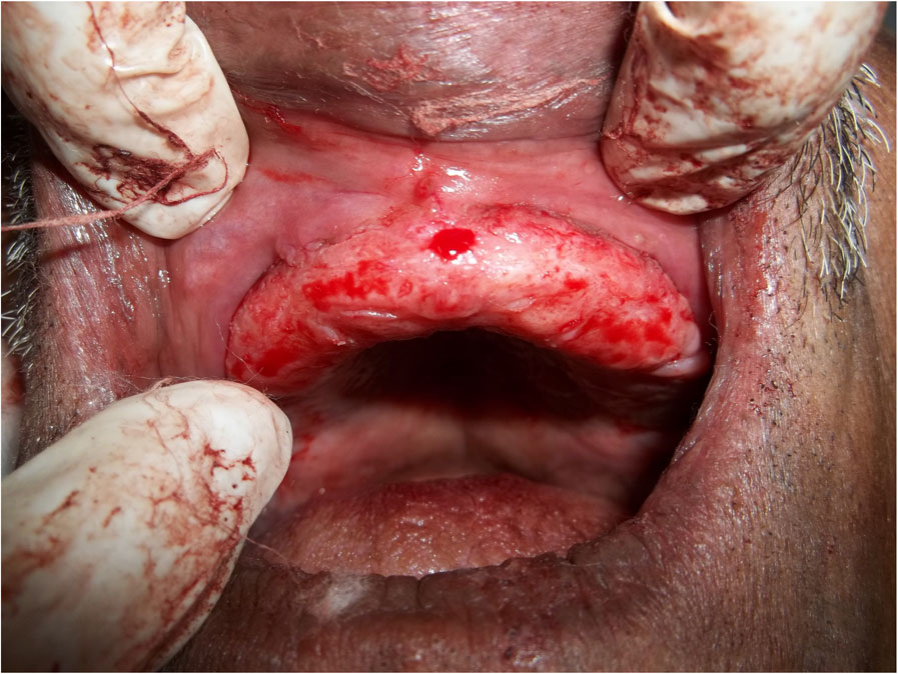
Excision of the tissue

**Fig. 5:**
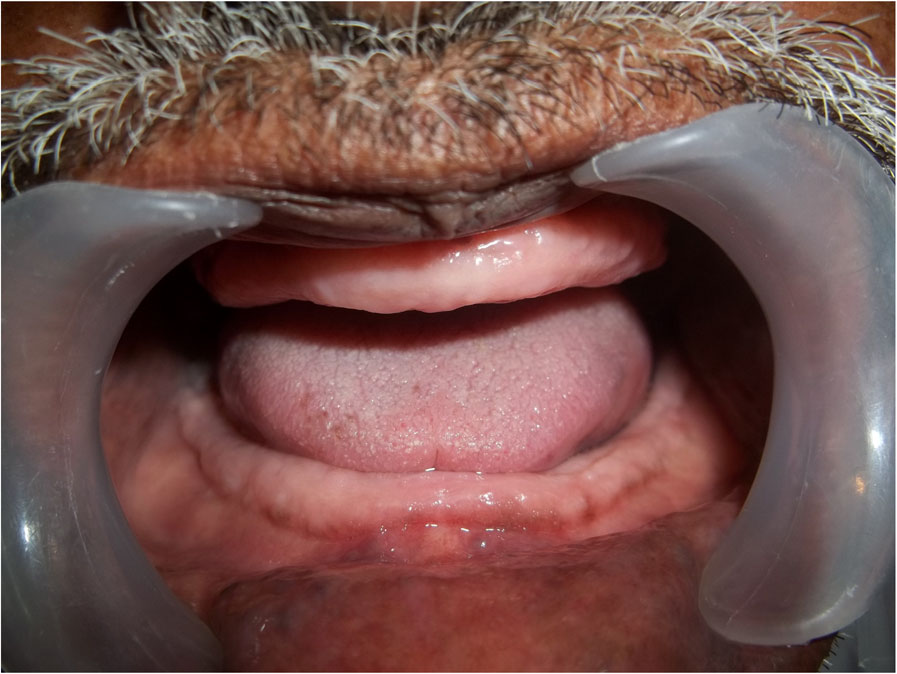
1 week post-operative follow up

**Fig. 6:**
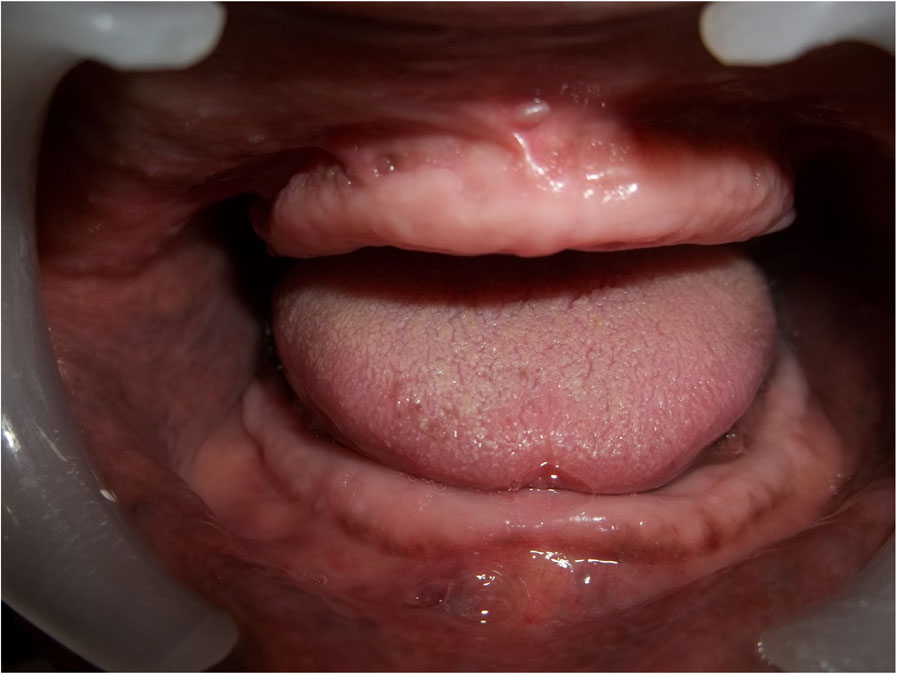
3 Months post-operative follow up

**Fig. 7:**
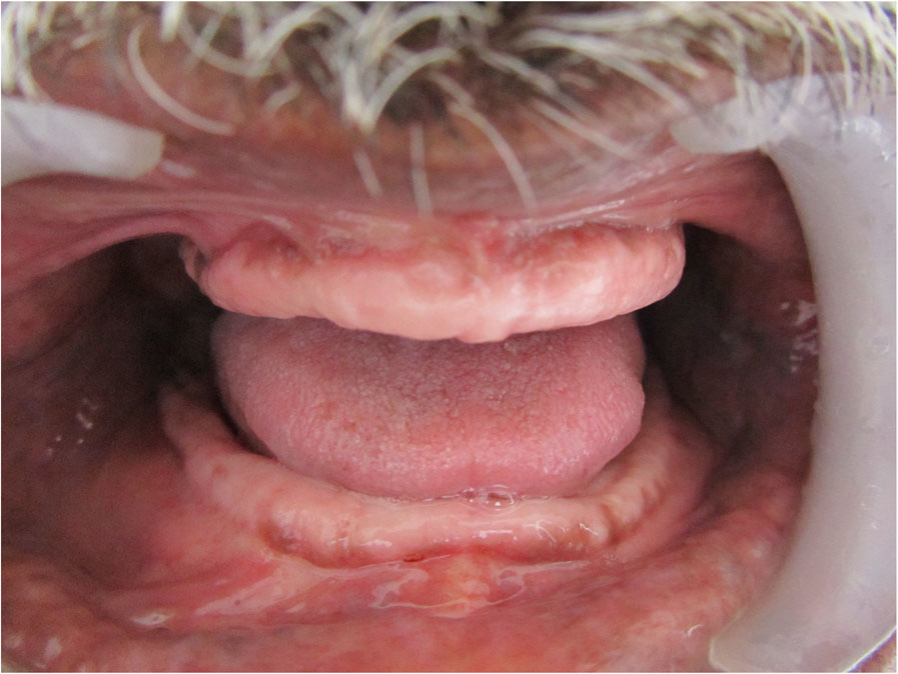
6 Months post-operative follow up

**Fig. 8:**
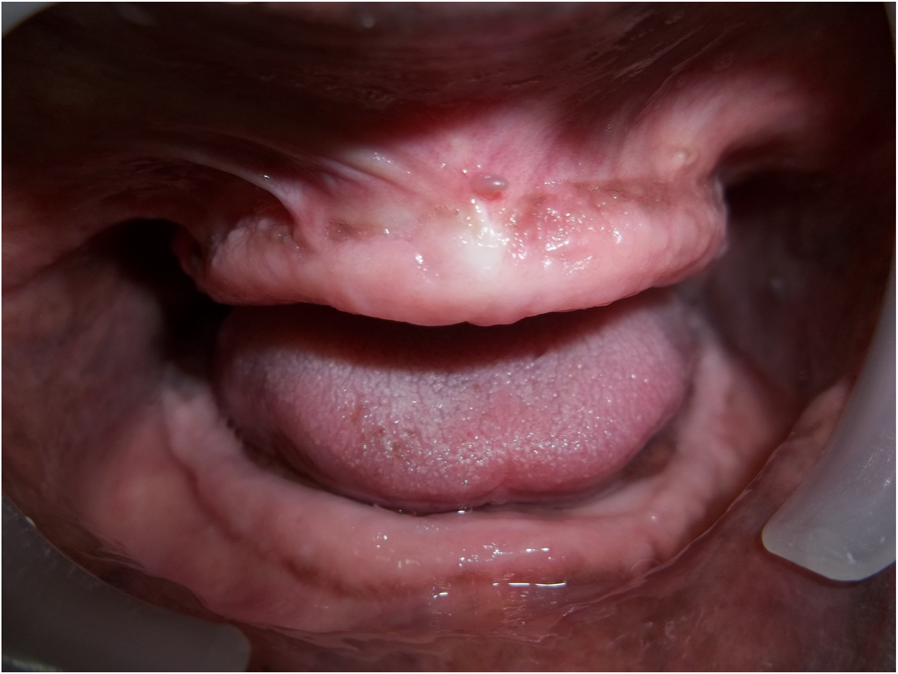
1 Year post-operative follow up

A 70-year old male patient reported to clinic with a chief complaint of swollen gums of 5 year duration and wanted to replace his missing teeth. Patient noticed swollen gums prior to his 4 years of edentulous state and the condition persisted to present. He was a known hypertensive and was on medication for the same since 7 years (10 mg Nifedipine/day).On intraoral examination- pink, firm, irregular, nodular, non- tender enlargements were found on labial aspects of maxillary and mandibular residual alveolar ridges. The enlargements were asymptomatic in nature with no history of bleeding. Both arches were completely edentulous (Fig. 1). Panoramic radiograph showed no osseous deformities of maxilla and mandibular ridges (Fig. 2). Patient’s complete blood count, bleeding time, clotting time and platelet count were with in normal limits. An incisional biopsy was obtained from anterior right side of the maxilla. Histological report revealed hyperplastic and acantholytic stratified squamous epithelium with elongated rete ridges extending into connective tissue which was fibro collagenous and showed focal areas of fibrosis. Infiltration of chronic inflammatory cells and congested blood vessels were seen which suggested of gingival hyperplasia (Fig. 3). As patient wanted complete dentures, we planned to surgically excise entire overgrowth. Considering medical status of the patient, drug was not altered because, dose taken by the patient was low (10 mg/day). Local anesthesia devoid of vasoconstrictor was used to remove fibrous tissue from alveolar ridge with help of 15 no. B.P blade (Fig. 4). To avoid any discomfort during the early phase of wound healing, a surgical splint was placed on both ridges after thoroughly covering the operated site with periodontal dressing (Coe Pack). The patient was recalled and followed at a period of 1 week, 3 months, 6 months and 1 Year intervals (Figs. 5, 6, 7 and 8). No recurrence of growth was observed during any of the recall visits. After 3 months of surgery, patient had his complete dentures fabricated. Even on further recall visits there was no recurrence of growth noticed.

## Discussion & conclusion

Nifedipine is a very potent and effective anti-hypertensive drug. However, long term use of these anti-hypertensive drugs causes gingival enlargement. In a community-based study, it was noticed that more than 6% of subjects taking Nifedipine had significant overgrowth and it was directly proportional to amount of gingival inflammation [3]. As suggested by Seymour eat al [8] drug induced gingival hyperplasia is a multifactorial disease. Gingival enlargement in our case persisted even in edentulous state. It might be due to persistence of gingival overgrowth which did not resolve completely after extraction or might be due to incorporation of specific population of gingival fibroblast in alveolar ridge mucosa [9]. The other reason for gingival enlargement in edentulous state can be due to defective collagen activity or due to decreased uptake of folic acid, blockage of aldosterone synthesis from adrenal cortex and an increase in adreno corticotropic hormone (ACTH) level and up regulation of keratinocyte growth factor [10]. Drugs like Nifedipine, block influx of calcium ions thereby affecting homeostasis of collagen. Synthesis and degradation of collagen being altered leads to the abnormal growth [11].Also a link to androgen metabolism has been suggested. Evidence from animal studies confirms that, nifedipine when added to gingival fibroblast in culture, increase the conversion of testosterone to 5α dihydrotestosterone and this active metabolite would target subpopulations of fibroblasts [12, 13]. Idiopathic/Hereditary gingival enlargement from our case was ruled out as these enlargements are commonly detected at an early age and in few cases even at birth. Histological findings of present case suggested drug induced gingival enlargement. Genetic factors like Polymorphism of enzymes that are involved in transport (P-glycoprotein MDR1, CYP2C) and metabolism (cytochrome P450) of pharmacological active substances have been investigated in various studies. A relationship has been described between gingival enlargement and the expression of human leukocyte antigen (HLA; HLA-DR2-positive patients) [14]. Other factors like heparin sulfate glycosaminoglycan (HSPG), basic fibroblast growth factor (bFGF), and transforming growth factor – beta (TGF-β) were found to be high in drug induced gingival enlargement [10]. Dose of drug in present case was not altered as the dose was very low. Several studies in literature have suggested that a dose range of 30-60 mg/day is more associated with gingival enlargement [3]. Dose of Nifedipine taken by patient was below the threshold limit of gingival overgrowth. Relation between gingival hyperplasia and pharmacokinetics of the drug has been investigated and are much debatable. This threshold might differ from patient to patient which might not be a suitable prognostic factors for gingival enlargement [8]. Normal ridges were noticed after surgical excision in our patient. There was no recurrence of growth on a year follow up and on regular use of denture by our patient.

In conclusion, Nifedipine induced gingival enlargement is rare to occur in edentulous patients as there are no such reported cases from the past. The possible etiology for its occurrence is obsolete. Further studies are required to explain the association and existence of Nifedipine induced gingival enlargement in edentulous patients. Hence, there is a need for physicians and dentist to make a coordinated treatment plan and practice care while prescribing these drugs which are associated with gingival overgrowth.

## Abbreviations

ACTH: Adreno corticotropic hormone
bFGF: Basic fibroblast growth factor
CCBs: Calcium channel blockers
HSPG: Heparin sulfate glycosaminoglycan
TGF-β: Transforming growth factor – beta
